# Managing Tsetse Transmitted Trypanosomosis by Insecticide Treated Nets - an Affordable and Sustainable Method for Resource Poor Pig Farmers in Ghana

**DOI:** 10.1371/journal.pntd.0001343

**Published:** 2011-10-11

**Authors:** Burkhard Bauer, Bettina Holzgrefe, Charles Ibrahim Mahama, Maximilian P. O. Baumann, Dieter Mehlitz, Peter-Henning Clausen

**Affiliations:** 1 Institute for Parasitology and Tropical Veterinary Medicine, Freie Universität Berlin, Berlin, Germany; 2 International Animal Health, Freie Universität Berlin, Berlin, Germany; 3 Veterinary Services, Pan African Tsetse and Trypanosomiasis Eradication Campaign (PATTEC), Accra, Ghana; Foundation for Innovative New Diagnostics (FIND), Switzerland

## Abstract

An outbreak of tsetse-transmitted trypanosomiasis resulted in more than 50% losses of domestic pigs in the Eastern Region of Ghana (source: Veterinary Services, Accra; April 2007). In a control trial from May 4^th^–October 10^th^ 2007, the efficacy of insecticide-treated mosquito fences to control tsetse was assessed. Two villages were selected – one serving as control with 14 pigsties and one experimental village where 24 pigsties were protected with insecticide treated mosquito fences. The 100 cm high, 150denier polyester fences with 100 mg/m^2^ deltamethrin and a UV protector were attached to surrounding timber poles and planks. Bi-monthly monitoring of tsetse densities with 10 geo-referenced bi-conical traps per village showed a reduction of more than 90% in the protected village within two months. Further reductions exceeding 95% were recorded during subsequent months. The tsetse population in the control village was not affected, only displaying seasonal variations. Fifty pigs from each village were ear-tagged and given a single curative treatment with diminazene aceturate (3.5 mg/kg bw) after their blood samples had been taken. The initial trypanosome prevalence amounted to 76% and 72% of protected and control animals, respectively, and decreased to 16% in protected as opposed to 84% in control pigs three months after intervention. After six months 8% of the protected pigs were infected contrasting with 60% in the control group.

## Author Summary

Sixty million people and more than 70 million livestock live in Africa at risk of contracting trypanosomiasis. The heads of member states of the African Union (AU) declared the year 2000 as the beginning of the Pan African Tsetse and Trypanosomiasis Eradication Campaign to eradicate tsetse flies and the diseases they transmit from the continent. For the first time the social and economic consequences of trypanosomiasis were brought to the attention of the affected populations. Efforts to control the fatal disease in man and livestock are based on treatment of patients and livestock with trypanocidal drugs. Resistance-related drug failures are increasing. Methods to control tsetse flies rely on insecticides. Past tsetse campaigns proved unsustainable due to the public good character of most control techniques such as aerial and ground spraying, traps or targets. Treating livestock with insecticides may be more sustainable and is also controlling ticks, which can transmit economically important and often fatal diseases. Costs per head of livestock and tick resistance against insecticides are seen as a major hindrance to their continuous large-scale use. Insecticide treated nets proved an effective and affordable means protecting pigs against tsetse transmitted trypanosomoses in Ghana.

## Introduction

The tsetse and trypanosomiasis problem to man and his livestock lies at the heart of Africa's poverty. Estimates of gross national per capita income show that 20 of the world's 25 poorest countries are affected by tsetse-transmitted trypanosomoses [1]. An ever-growing demand for meat and milk products has led to a policy where improvements in productivity are sought by introducing exotic breeds or by cross-breeding pure-bred with indigenous animals. Human and agricultural activities may lead to the disappearance or reduction of savannah tsetse species notably in West Africa, but riparian tsetse flies, particularly so in the more humid regions still persist in large numbers, even in peri-domestic sites. Disease risks and poverty have not been reduced over the last 30–40 years in much of rural West Africa, leading to an increasing migration of the affected populations to the large urban centres. PATTEC activities are presently implemented in six out of 37 affected countries. Lack of funding and the complexity of the endeavour, particularly in the vast rainforest areas, may lead to slower progress than originally anticipated. Therefore, improving food security and income of resource-poor farmers in these areas needs to be urgently addressed. Income generating activities for rural populations are invariably linked to an improvement of animal production, which is still threatened by the presence of tsetse flies and other arthropod vectors.

**Figure 1:**
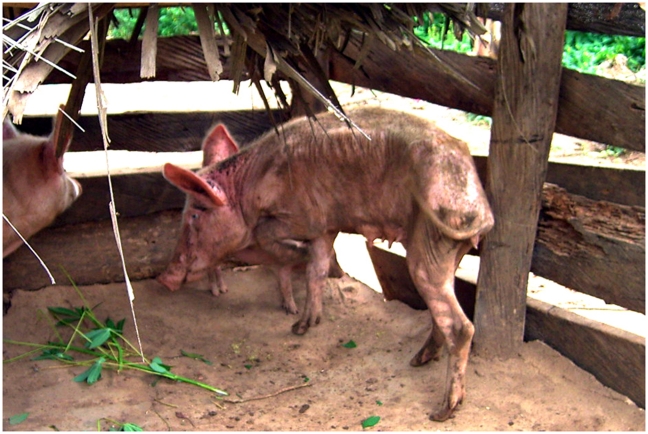
Emaciated trypanosome positive sow, Eastern Region, Ghana.

The occurrence of vector-borne diseases calls for continuous veterinary surveillance and considerable investments in drugs for animal health care. Keeping valuable stock confined to pens (zero-grazing scheme) is a strategy to reduce the risk of tick-borne diseases. It also reflects changes in livestock husbandry management systems such as an increasing scarcity of grazing resources and the need to enhance productivity. However, tsetse flies and other insect vectors can enter the pens, feed on confined stock and transmit African animal trypanosomiasis (AAT). Protection of valuable livestock with an insecticide-treated mosquito net (ITN) significantly reduced the risk of vector-borne diseases as was shown in Western Kenya [2]. Pigs are an essential source of income and for household consumption in rural areas of the Eastern Region of Ghana but the persisting threat of AAT may at any time jeopardize a productive pig husbandry (Fig. 1) as was experienced in a recent outbreak. Our intervention in the Eastern Region of Ghana was based on the hypothesis that surrounding pigsties with ITN would result in a reduction of tsetse flies, thereby also reducing the risk of trypanosome infections. Thus, we set up a trial with the objectives to recording baseline data on tsetse fly dynamics and, simultaneously estimating the risk of trypanosome infections in pigs in an intervention and control village before and after installation of ITN.

## Materials and Methods

**Figure 2:**
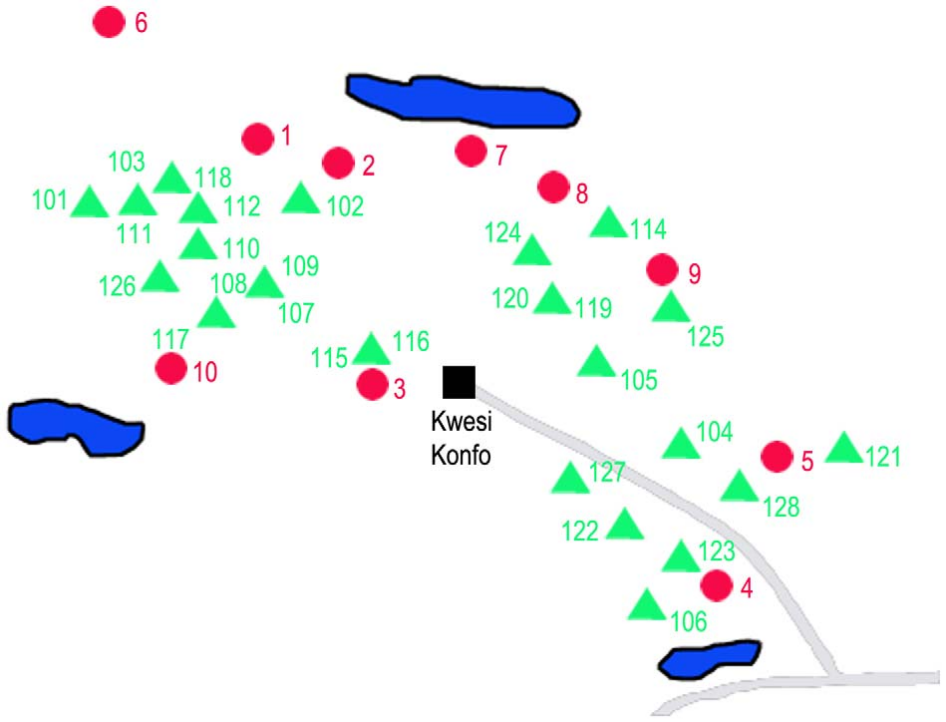
Map of Kwesikonfo, Eastern Region; intervention village. Trap locations No. 1-10 (red), near pigsty locations No. 101–128 (green).

**Figure 3:**
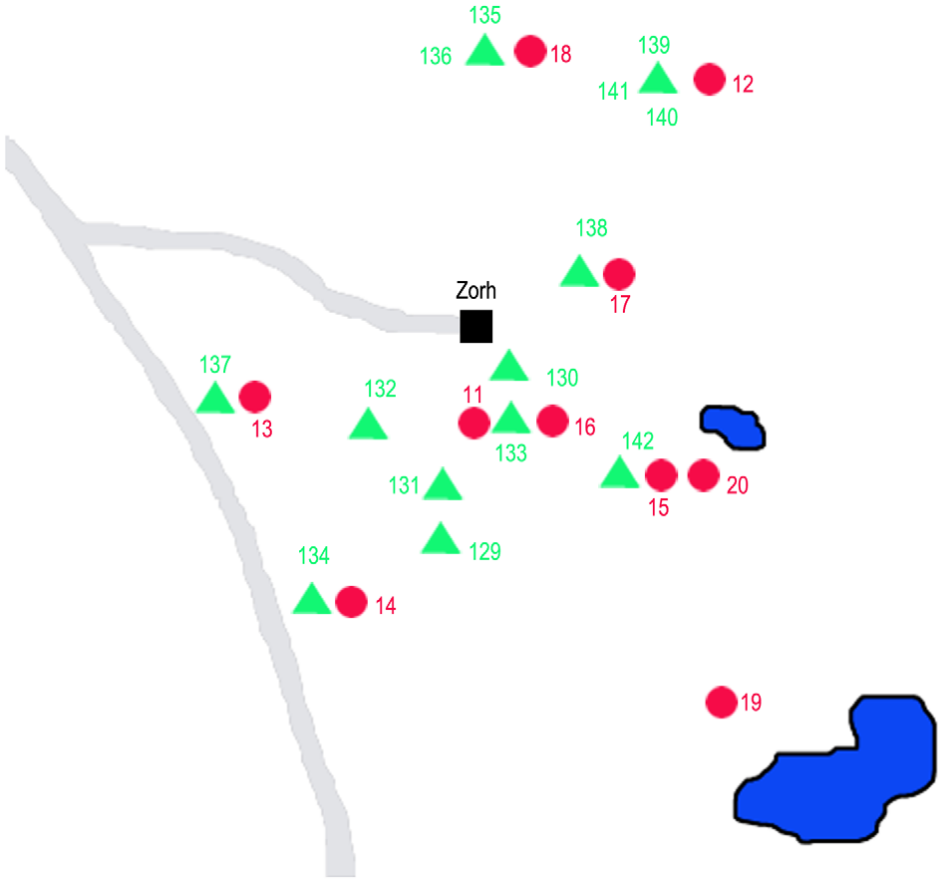
Map of Zorh, Eastern Region; control village. Trap locations No. 11-20 (red), near pigsty locations No. 129–142 (green).

The two selected trial sites were located in the district of Suhum/Kraboa/Coaltar, Eastern Region, Ghana (Fig. 2 and 3) where the national veterinary services had recently identified trypanosomiasis as the leading cause for sudden pig mortalities during April 2007. The villages were comparable in animal husbandry management systems, livestock composition and numbers. Pigs are confined to pens constructed of local timber. Given their small size piglets sometimes succeed to leave, thus, freely roaming in the vicinity of their pigsties. More than 90% of the pig breeds are crossbred large white. Both villages are situated in areas with a variety of water courses and marshy habitats. The vegetation consists of tropical seasonal rain forest mixed with a variety of locally cultivated crops such as cocoa, oil palm, cassava and plantain as well as small plots of maize and sugarcane. Frequent rainfalls and the dense vegetation provide favourable conditions for the tsetse flies. The two villages are mere islands in a sea of dense, fringing tropical rainforest. Heavy rainfall followed by periods of intensive sunshine is characteristic of the region with temperatures ranging between 22–32°C and a relative humidity often attaining or exceeding 90%. The longer of the two rainy seasons lasts from May – mid-July and the second, shorter one from September – mid-October.

Kwesikonfo, experimental village, is located N 6° 33′W 0° 33′ and had 27 pigsties of which 24 were protected with fence material. The 111 pigs consisted of one boar, 15 sows, 53 growers and 42 piglets (Fig. 2). Zorh is located N 5° 59′W 0° 21′ serving as control village, with 14 farmers participating in the trial (Fig. 3). The pig population in Zorh consisted of 106 pigs; 8 boars, 24 sows, 13 growers and 61 piglets. The distance between the two villages is approximately 30 km.

**Figure 4:**
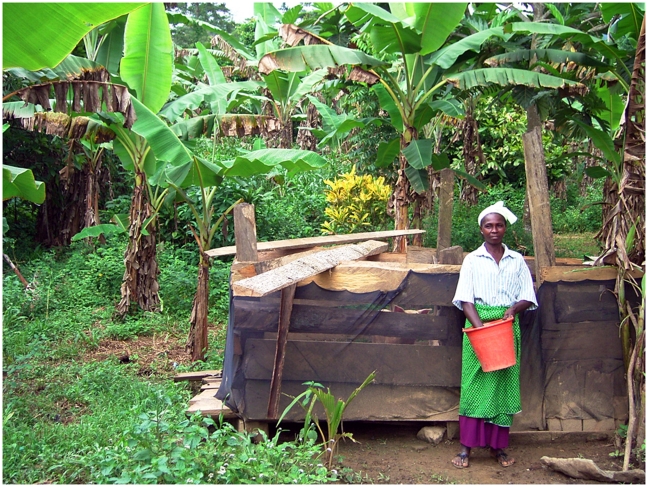
Pigsty protected with insecticide-treated net in Kwesikonfo.

Based on previous experience we deployed, as in Kumasi [3], 1 meter high ITN (Provision from Vestergaard Frandsen Disease Control Textiles) consisting of black multi-filament polyester with a mesh width of 1×2 mm, pre-treated with deltamethrin (100 mg/m^2^) and a UV-protector for enhancement of the persistency of the insecticide. The ITN had a reinforced hem and was attached to the pigsties with one inch nails (Fig. 4). Whenever the nets became soiled the farmers were instructed to clean the material with a soft brush, thus ensuring better availability of the insecticide on the surface. Animal movements or farmers' manipulations sometimes resulted in fence damage which was subsequently repaired by patches of netting material. Some farmers used chicken wire to reinforce their net. Apart from repair of damages the ITN remained in place for six months.

Monitoring of the insecticide persistency was ensured by monthly collections of ITN samples from 13 different farms. Fifty – sixty laboratory reared *Musca domestica* were used for monitoring. Duration of 10 sec in a test box (Patent No. 08803541.5 – 1260 PCT/EP 2008061570) of which the interior had been coated with the respective ITN sample was considered as the minimal time of contact for *M. domestica* colliding with the net under field conditions. This had been confirmed during on-farm surveys [4]. After their passage in the test box the flies were released into an observation cage allowing recording of subsequent paralysis as well as its speed and duration for a time span of 6 h. According to our own observations house flies are similar to tsetse flies in their susceptibility. Fifty laboratory-reared female *Glossina palpalis gambiensis* in the late stage of their third reproductive cycle were all paralyzed within 5 min following an exposure of 10 sec in the test box (Bauer, unpublished data). Previous work has shown that most of the tsetse flies will subsequently perish as a consequence of their prolonged paralysis lasting for or exceeding 6 h – either due to predation or desiccation [5]. Control flies were exposed in another, untreated test box and subsequently released for further controlling into another observation cage.

The tsetse fly densities were monitored every two weeks for 48 h throughout the trial period with 10 unbaited, georeferenced bi-conical traps per village. The selected trap locations were always outside of, but close (about 5–20 m) to the pigsties or watering places (Fig. 2 & 3). Any vegetation surrounding the traps was cleared ensuring an optimal visibility. Grease was applied to the poles, preventing ants from entering the cages. The cages containing tsetse were stored in a cool box covered with a wet towel ensuring optimal transport conditions. All tsetse catches were recorded, separated into males and females. The physiological age of males was determined through wing fray analysis [6].

At the onset of the study blood samples were collected from the jugular vein from all ear-tagged pigs before receiving a blanket trypanocidal treatment (diminazene aceturate, 3,5 mg/kg bw). Thin blood smears were dried and preserved with 96% ethanol and stained with Giemsa's solution before examination for trypanosome infections with a compound microscope (10×100 oil immersion).

Data were processed in MS Excel to prepare x-y-z graphs for fly distributions. As fly catches per trap and day do not follow a normal distribution, data were subjected to a natural log transformation with subsequent least square regression to illustrate trends over time;

Chi square and Fisher's exact tests were used to determine significant differences in trypanosome infection rates before and after intervention [7].

### Ethics Statement

The study protocol followed the appropriate technical requirements, procedural instructions and the principles of Good Clinical Practice as outlined in the “International Co-operation on Harmonisation of Technical Requirements for the Registration of Veterinary Medicinal Products (VICH)” guideline CVMP/VICH/595/98 “VICH TOPIC GL9 Step 7 –Guideline on Good Clinical Practices (CVMP approved July 2000).”

The study protocol was presented to, discussed with and approved by the national authorities (Ghanaian Veterinary Services).

The study protocol was presented to all animal owners who gave their consent to their involvement.

## Results

### Tsetse catches

Two pre-intervention catches were conducted on May 4^th^ and 9^th^ 2007. After protection of the pigsties and in comparison with the initial numbers, the overall catches in Kwesikonfo were reduced by more than 95% from the 3^rd^ survey onwards except one catch (5^th^ survey) where 11.9% were recorded. *Glossina palpalis palpalis* was the only tsetse species detected.

**Figure 5:**
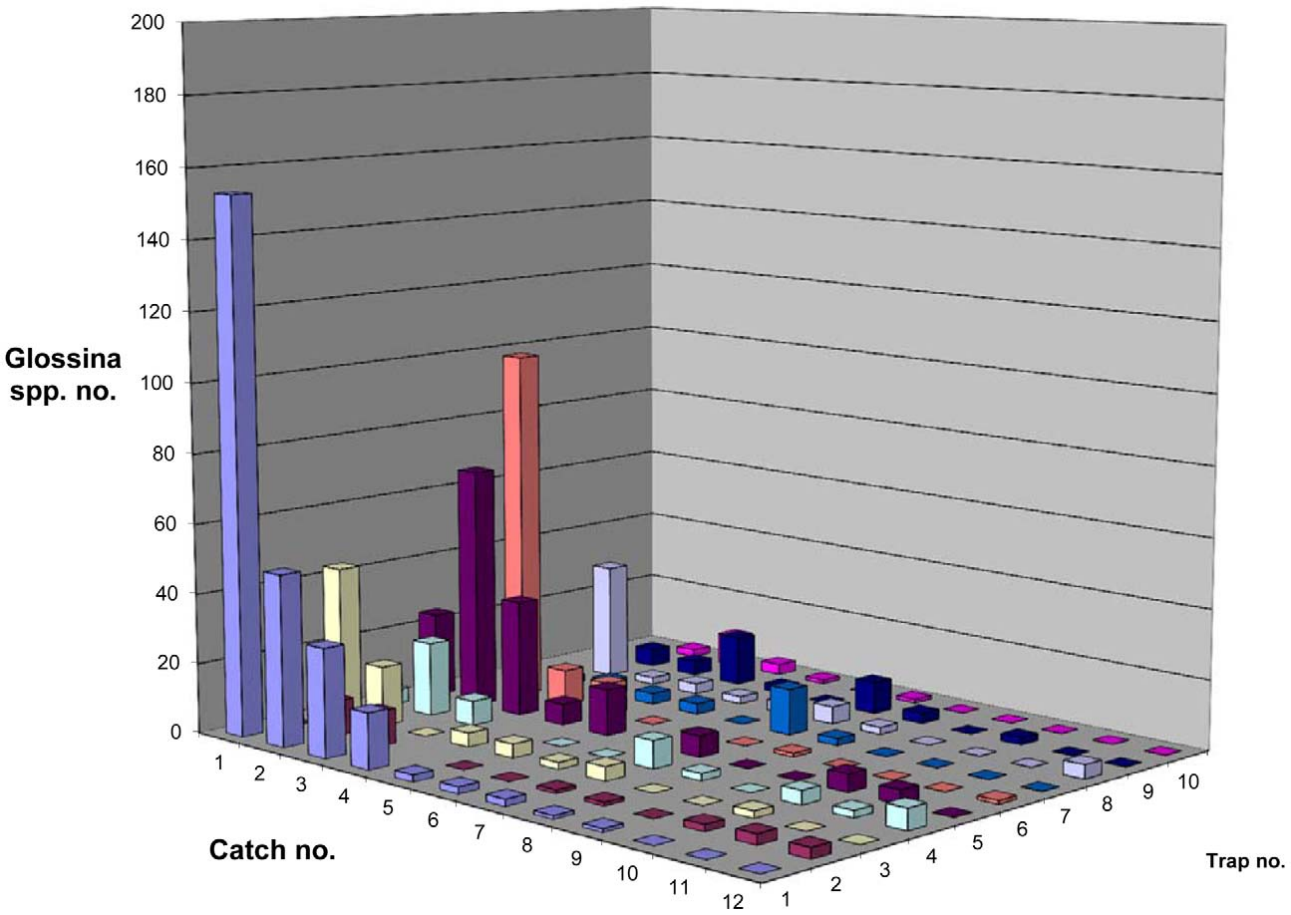
Catches of *Glossina palpalis palpalis* with 10 geo-referenced traps in Kwesikonfo Village (24 pigsties protected with ITN). Catch no. 1 and 2 represent the tsetse population at the start of the study, catch no. 12 after the experimental period.

**Figure 6:**
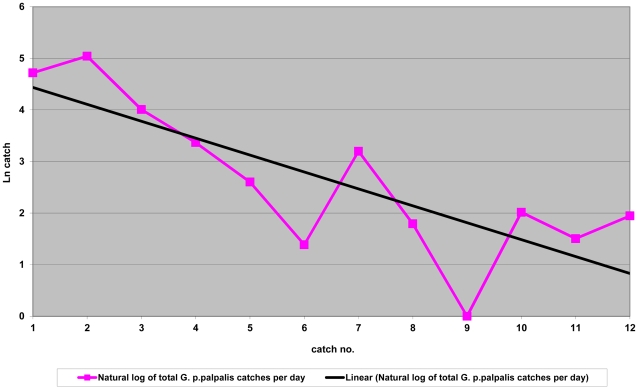
Natural log transformation of total *G. p. palpalis* per day over 12 catches in Kwesikonfo Village. Evolution of catches before (surveys 1 and 2) and after protection (no. tsetse/traps/day). Least square regression: y = 4.7−0.328×(p = 0.002, R^2^ = 0.634), significant.

Fig. 5 also shows that all catches declined regardless of the trap position. In the absence of any further intervention or a barrier against tsetse flies, a continuous reinvasion from the adjacent tropical rainforest could be expected. Despite this external population pressure we recorded a significant decline of the tsetse population as shown in Fig. 6 (natural log-transformation).

**Figure 7:**
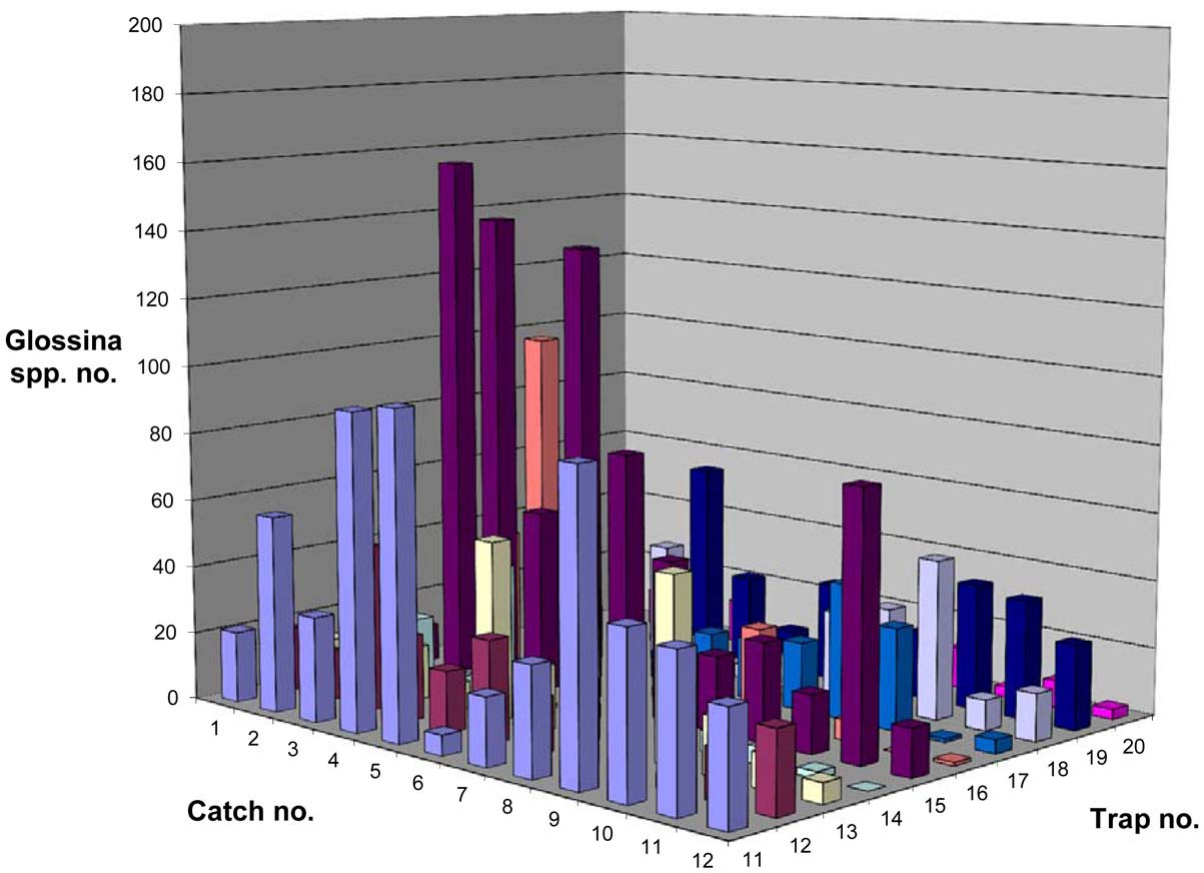
Catches of *Glossina palpalis palpalis* with 10 geo-referenced traps in Zorh Village (control village, 14 unprotected pigsties). Catch no. 1 and 2 represent the tsetse population at the start of the study, catch no. 12 after the experimental period.

**Figure 8:**
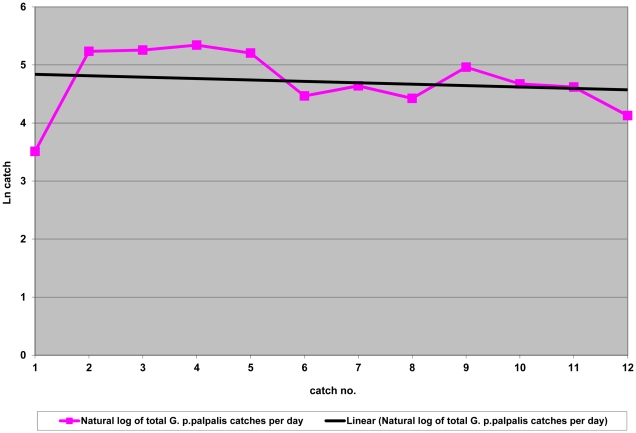
Natural log transformation of total *G. p. palpalis* per day over 12 catches in Zorh Village (control village). Evolution of catches (no. tsetse/traps/day) in the absence of intervention. Least square regression: y = 4.7−0.025×(p = 0.62, R^2^ = 0.026), not significant.

At the same time, the tsetse catches in Zorh (control village) remained largely unchanged as shown in Fig. 7 and 8 (natural log-transformation). Catch reductions between 30–48% were however observed on three occasions and were attributed to heavy rainfall during the respective surveys in the control village, which resulted in a decrease of flight activity.

The results of the wing fray analysis show similar proportions of the younger age categories (teneral males, wing fray 1) between the villages before intervention, with a proportion of 18–24%, respectively, of the younger flies. After intervention this proportion increased to more than 70% in the intervention village as opposed to slightly more than 40% in the control village. The proportion of older flies (wing fray 2 or more) dropped from more than 65 to 25% of all flies in the intervention village but remained at almost 60% in the control village.

### Trypanosome prevalence

The examination of thin blood smears revealed a high prevalence of trypanosomes belonging to the subgenus *Nannomonas*, amounting to 76% (intervention) and 72% (control) at the onset of the study (Table 1). The treatment of all ear-tagged animals resulted in a significant decline of the prevalence in the intervention village to 16%; this contrasted with the results of the control village, showing an increase to 84% at the same time. After six months only 8% of the protected pigs were trypanosome positive as compared with 60% infections in the control pigs.

**Table 1:** Trypanosome prevalence in Kwesikonfo (protected) and Zorh (control) villages before and after intervention.

	Kwesikonfo	Zorh
Trypanosome infection rate	p (95% Confidence Interval)	
Before intervention	0.76^a^ (0.578, 0.762)	0.72^a^ (0.632, 0.808)
3 mths after intervention	0.16^b^ (0.088, 0.232)	0.84^a^ (0.768, 0.912)
6 mths after intervention	0.08^b^ (0.027, 0.133)	0.60^b^ (0.504, 0.696)

a,b Superscript with different letter denote significant difference at p<0.05 level.

### Measuring persistence of ITN with bioassays

Over the experimental period we observed an increase of time between exposure and paralysis of flies exposed to ITN. While exposure to samples from May–July resulted in 100% paralysis during the first 5–10 min, it took 30–120 min (last samples from October) before 100% paralysis were reached at the termination of the study.

## Discussion

### Significant impact of ITN on tsetse flies

Our results show that the protection of pigs with ITN significantly reduced tsetse fly numbers and subsequently trypanosome prevalence. *G. p. palpalis* was the only species caught. It is predominant in the Eastern Region of Ghana, sometimes even displaying endophagic behaviour, for instance, by attacking pupils while attending classes. This particular behaviour was subsequently confirmed by staff from the Veterinary Services who caught tsetse flies inside classrooms. The perception of success when using ITN not only points to the private but also to the public good character of our approach since the marked reduction of tsetse flies in the whole village area was well observed and appreciated by the villagers as additional benefit. In peri-domestic and domestic habitats tsetse flies of the *palpalis* group are mainly feeding on man and *suidae*. As such they are the principal vectors of *T. brucei gambiense* in West and Central Africa [8]. More recently pigs were found to harbour *T. brucei rhodesiense* infections in Western Kenya [9]. Pig breeding can thus be considered as an important risk factor for spreading *gambiense* and now *rhodesiense* sleeping sickness [9], [10], particularly as pigs were incriminated as main non-human hosts of the disease [8], [10]. Therefore, continuous vector control has been suggested more recently as an effective means for reducing the human sleeping sickness risk [11]. Our results indicate that the use of ITN in areas with endemic sleeping sickness is likely to have a rapid effect on the disease risk if pigsties can be protected as described here.

### ITN kill other nuisance insects and maybe mosquitoes

ITN of 1 m height successfully protected bulls against attacking nuisance insects in Kumasi, Ghana. As a result their defensive movements were significantly lower (by more than 80%) in comparison to unprotected controls. Catches of nuisance insects at distances of 20–30 m outside the protected pen were also significantly (70%) reduced [3]. House and stable flies are synanthropic pests and have a world-wide distribution. They can mechanically transmit several disease agents, including *Trypanosoma* spp. by stable flies [12]–[14]. Continuous disturbance by biting flies may even turn out to be more important since it distracts the animals from feeding and resting, leading to reductions in average daily gains [15]. Many mosquito species are displaying anthropophagic as well as zoophagic behaviour [16], [17]. Protecting livestock with insecticide-treated nets may thus have an effect on mosquito borne diseases. However, further in-depth studies are required before drawing far-reaching conclusions. Eventual effects of ITN on mosquito populations and other insects of medical importance are presently evaluated during several IFAD/FAO funded trials in selected sub-Saharan countries.

### ITN and the prospects of sustainability

The farmers also felt that they had a user-friendly technique at their disposal. All pigsties were protected by the farmers themselves, only supervised by staff from the Veterinary Services. At this stage we ignore whether the perceived benefits would translate into farmers' willingness to purchase ITN for protection of their livestock. However, at a price of US $ 0.90 per m^2^ the annual costs for protecting an average pigsty of 10–15 m perimeter would amount to 10.0–15.0 US $. The calculation for 1 m of ITN is based on 1 US $/m^2^ if the necessary material for their attachment is included. With a persistency exceeding 8 months in a comparable climate [3] it would be necessary to replace the ITN annually. A total of 340 m was required to protect 24 pigsties in an area of about 10 km^2^; hence costs per km^2^ amount to 34.0 US $. Detailed costing of available tsetse control techniques was compiled in two studies [18], [19] at a stage when ITN had not yet been introduced. At first sight ITN may offer an attractive, user-friendly and affordable alternative to the existing control methods. Therefore an update of actual costs for the now available techniques is clearly warranted.

Recent trials have shown that a blue-and-black panel of cloth flanked by a panel of fine insecticide-treated black netting of just 0.12 m^2^ was effective in controlling *G. f. fuscipes* [20]. This should lead to a reduction of costs. But both costs and efficacy of this method remain to be confirmed and compared with the approach we are presenting here, where the prime objective was to effectively protect confined pigs. ITN could overcome the problem of sustainability. In protecting the participating farmers' livestock they constitute a private good with a public good character. These externalities are likely to accrue; not only most tsetse but also other nuisance and biting flies colliding with the net will be killed, thus benefiting all villagers and their livestock [3]. Hence, maintenance of livestock and income generation in areas deemed unsuitable because of the tsetse related trypanosomiasis risk has become a viable option.

### Impact of ITN on trypanosome prevalence

The trypanosome prevalence was significantly reduced in protected pigs whereas it remained much higher in the control animals. Parasitological examination of thin blood smears revealed trypanosomes belonging to the subgenus *Nannomonas*, represented by relatively small forms, without a free flagellum, a rounded posterior end and a subterminal and medium sized kinetoplast. High and sudden mortalities in pigs are generally attributed to acute or per-acute infections with *T. simiae* [21], [22]. The clinical symptoms of the disease and also the *post mortem* findings very much resemble the observations made by the Ghanaian Veterinary Services before trial inception. No trypanosome isolates exist from this particular outbreak. Work from other authors indicates the likely presence of mixed infections of trypanosomes of subgenus *Nannomonas* [23]–[26].

### Environmental impact of ITN

In our case 340 m of ITN were sufficient to protect 111 pigs in 24 pigsties. If 1 m of net contains 100 mg/m^2^, 34 g of deltamethrin would suffice to significantly reduce tsetse flies in a village of 10 km^2^. Protecting livestock with ITN in Germany was evaluated and seen as environmentally acceptable method due to its targeted and limited insecticide application. No traces of insecticide were detected in meat, milk and water and only negligible amounts were found in soil samples. Some insect species (hovering flies, *Syrphidae*) and Hymenoptera are considered indicator insects. Catches with Malaise traps revealed that these species were not or only marginally (*Syrphidae*) affected. [27].

The use of ITN has led to significant improvements in an area which is offering particularly favourable conditions for the survival of large tsetse fly numbers resulting in a high risk of trypanosome infections. The technique has proved effective and user-friendly. For its further dissemination it is necessary to create public awareness of its considerable potential in tsetse and trypanosomiasis-afflicted areas. More technical knowledge, particularly with regard to the effects of ITN in tsetse free areas against other medically relevant flies and mosquitoes, is clearly warranted. Anthropological studies may help to embody this technique as a promising tool for the control of relevant insect vectors and nuisance flies in improved farming systems.
